# ‘Errors’ and omissions in paper-based early warning scores: the association with changes in vital signs—a database analysis

**DOI:** 10.1136/bmjopen-2014-007376

**Published:** 2015-07-03

**Authors:** David A Clifton, Lei Clifton, Dona-Maria Sandu, G B Smith, Lionel Tarassenko, Sarah A Vollam, Peter J Watkinson

**Affiliations:** 1 Institute of Biomedical Engineering, University of Oxford, Oxford, UK; 2 Centre for Statistics in Medicine, University of Oxford, Oxford, UK; 3 Centre of Postgraduate Medical Research & Education (CoPMRE), the School of Health & Social Care, Bournemouth University, Bournemouth, UK; 4 Nuffield Department of Clinical Neurosciences, University of Oxford, Oxford, UK

**Keywords:** Early warning system, Patient safety, vital signs monitoring

## Abstract

**Objectives:**

To understand factors associated with errors using an established paper-based early warning score (EWS) system. We investigated the types of error, where they are most likely to occur, and whether ‘errors’ can predict subsequent changes in patient vital signs.

**Methods:**

Retrospective analysis of prospectively collected early warning system database from a single large UK teaching hospital.

**Results:**

16 795 observation sets, from 200 postsurgical patients, were collected. Incomplete observation sets were more likely to contain observations which should have led to an alert than complete observation sets (15.1% vs 7.6%, p<0.001), but less likely to have an alerting score correctly calculated (38.8% vs 30.0%, p<0.001). Mis-scoring was much more common when leaving a sequence of three or more consecutive observation sets with aggregate scores of 0 (55.3%) than within the sequence (3.0%, p<0.001). Observation sets that ‘incorrectly’ alerted were more frequently followed by a correctly alerting observation set than error-free non-alerting observation sets (14.7% vs 4.2%, p<0.001). Observation sets that ‘incorrectly’ did not alert were more frequently followed by an observation set that did not alert than error-free alerting observation sets (73.2% vs 45.8%, p<0.001).

**Conclusions:**

Missed alerts are particularly common in incomplete observation sets and when a patient first becomes unstable. Observation sets that ‘incorrectly’ alert or ‘incorrectly’ do not alert are highly predictive of the next observation set, suggesting that clinical staff detect both deterioration and improvement in advance of the EWS system by using information not currently encoded within it. Work is urgently needed to understand how best to capture this information.

**Strengths and limitations of this study**

- We undertook a large study of error rates when using a well-established early warning system, a widely adopted but relatively little studied healthcare tool.
- Alerts for patients becoming physiologically unstable were commonly missed when incomplete observation sets were taken—suggesting that this practice should be avoided.
- Alerts were also commonly missed when patients first became unstable, suggesting an important opportunity is being missed.
- ‘Incorrect’ alerts or ‘missed’ alerts were highly predictive of the next observation set, suggesting that work is needed to understand what additional information clinical staff use to ‘outperform’ the early warning system.
- The generalisability of our results may be limited by the single centre design restricted to surgical patients.

## Introduction

Paper-based early warning score (EWS) systems are a common part of the management of hospitalised adult patients. Such systems assign weightings to vital sign observations; these weightings are summed to produce an aggregate score. An alert may be generated if any individual weighting or the aggregate score are above set thresholds. Smith and Oakey^1^ investigated error rates in aggregate scores as they introduced an EWS during an outbreak of Legionnaires’ disease, reporting error rates >20%. Error rates appeared to change over time and a large proportion of observation sets did not have aggregate scores calculated, making the findings difficult to generalise. High error rates have also been found in the assignment of weightings^2^ and in a classroom environment when nursing staff entered vital signs from a provided list onto an early warning chart.^3^ Mohammad *et al*^4^ found that, even when using a handheld computer, error rates were increased on the ward in comparison to the classroom environment. Finally, error rates have been found to depend on the complexity of the scoring system.^5^

Despite these findings, there has been little investigation of the factors underlying errors when using an established EWS system in normal clinical ward practice. However, understanding these is critical for improving performance. The aims of this study were (1) to understand factors associated with errors when using an established paper-based EWS system, and (2) to investigate the types of error, where these are most likely to occur, and whether ‘errors’ can predict subsequent changes in patient vital signs. Therefore, using a large database of observations, we documented (A) errors in the assignment of weights to vital sign values, (B) errors in weight aggregation (summing the individual weights for each vital sign), (C) the effect of time of day on the occurrence of errors, and (D) how errors affected the clinical response that should have occurred. We examined the scoring pattern for observation sets before an error occurred, and whether an ‘error’ associated with an observation set predicted the true EWS for subsequent observations.

## Methods

We performed a secondary analysis of a large database of observations generated during the Computer ALerting Monitoring System 2 (CALMS-2) study (Mid and South Buckinghamshire Research Ethics Committee, REC No. 08/H0607/79). The CALMS-2 study was a before and after study to determine whether continuous monitoring of ‘vital signs’ with computer-modelled alerting to detect patient deteriorations reduced patients’ hospital length of stay. Paper-based EWS observation sets were collected from postsurgical patients, based in a specialist surgical ward within the Oxford University Hospitals NHS Trust. Patients meeting the inclusion criteria (see online supplementary appendix A) were recruited consecutively.

### Ward staff

The ward had between 20 and 28 beds in use throughout the study. A qualified nurse was responsible for the care of six or seven patients during night shifts, and four or five patients at all other times. Three clinical support workers were available during the early part of each day, while one clinical support worker was available at night. The normal ward staff undertook the majority of extrashifts where these were required. Ward staff were trained in the use of the EWS system when it was introduced or when they first started work on the ward using a structured training package developed by the hospital's Recognition of Acutely Ill and Deteriorating Patients committee. Annual updates occurred as part of the resuscitation training.

### EWS chart

**Figure 1:**
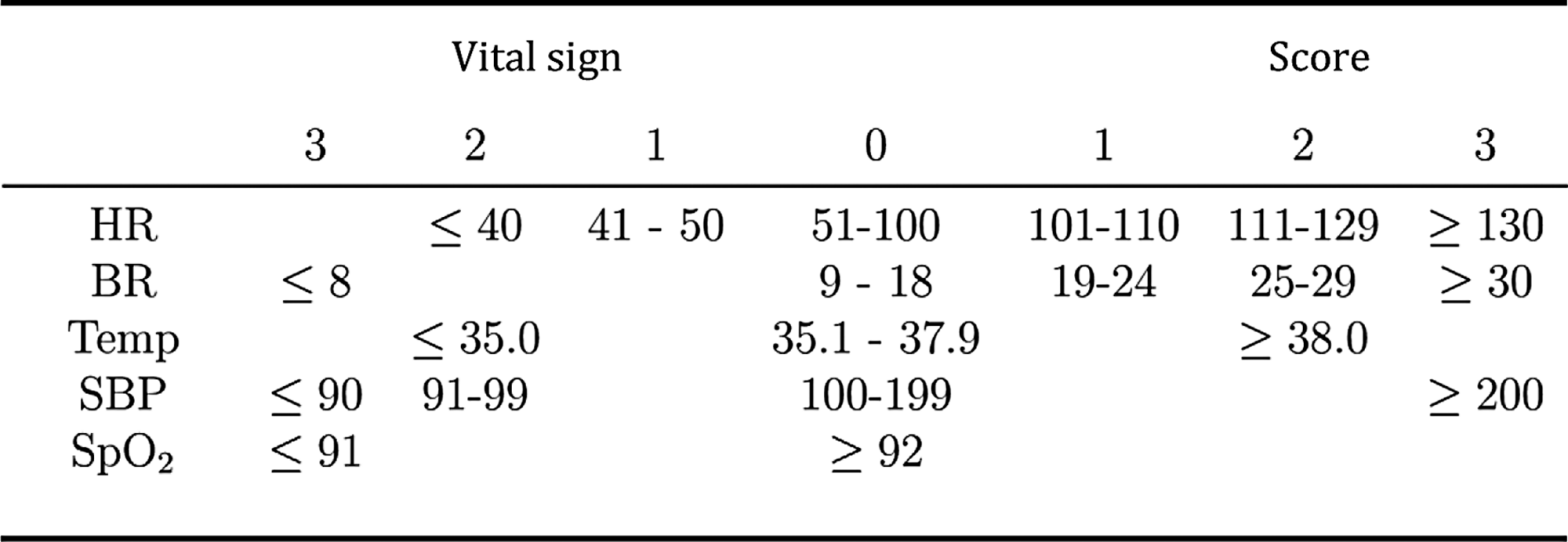
Early warning score (EWS) system used by clinical staff throughout the study, including heart rate (HR, measured in bpm), breathing rate (BR, measured in respirations per minute), temperature (Temp, measure in degrees Celsius), systolic blood pressure (SBP, measured in mm HG), and SpO_2_ (%).

The EWS used in the ward throughout the study has been in standard practice for more than a year before the study began (figure 1) (note: the escalation protocol is provided in online supplementary appendix B). An observation set was deemed to have ‘alerted’ if any single vital sign was assigned a weight of 3, or if the aggregate score was 4 or more.

### Data collection

Two electronic transcriptions of the paper-based EWS charts were performed. The first was performed by trained research nurses each weekday using all paper-based observation sets completed by ward staff from the preceding 24 h (or 72 h following a week-end). After the patient had been discharged, a research nurse, who had not seen the first transcription, performed a second transcription. The transcriptions comprising (1) vital sign values recorded by the ward staff, (2) weights assigned to each vital sign value by the ward staff and (3) the aggregate score recorded by the ward staff were stored in separate databases. The following rules were used to compare the two electronic transcriptions. Criteria 2–4 were required because these were graphically recorded vital signs, as marks on a paper chart. Ranges for criteria 2–4 correspond to graphically separated ranges for each vital sign.

1. If the values for the two transcribed observations would have resulted in different EWS weights being assigned to those values, then the two values were not averaged but were reviewed by a trained third expert.
2. If the heart rate values for the two transcribed observations were within 10 bpm, the mean of the two was taken—unless criterion 1 was met.
3. If the temperature values for the two transcribed observations were within 0.5°C, the mean of the two was taken—unless criterion 1 was met.
4. If the systolic blood pressure values for the two transcribed observations were within 10 mm Hg, the mean of the two was taken—unless criterion 1 was met.
5. The respiratory rate values for the two transcribed observations were only accepted if they were the same for both transcribed observations.
6. The SpO_2_ values for the two transcribed observations were only accepted if they were the same for both transcribed observations.
7. The EWS values (those assigned to each vital sign, and the summation EWS value) for the two transcribed observations were only accepted if they were the same for both transcribed observations.

Under all other circumstances, differences were resolved by a trained third expert, who made the final decision by reviewing the original paper-based recording. Transcription was done to the nearest digit, without rounding. Examples of reasons for differences between transcribed results are shown for interest (see online supplementary appendix C).

### Analysis and definitions

We sought to determine

1. The relationships between observation set completeness, alerts and errors
2. The effect of time of day on the occurrence of errors
3. The effect of the aggregate score sequence on error rates
4. The types of errors in complete observation sets
5. The relationship between ‘incorrect’ aggregate scores and the score of the subsequent observation set

We documented errors in the assignment of weights to vital sign values, errors in weight aggregation, the effect of time of day on the occurrence of errors, and how errors affected the clinical response that should have occurred. We examined the scoring pattern for observation sets before an error occurred and whether an ‘error’ associated with an observation set predicted the true EWS for subsequent observations.

Comparisons of proportions were performed using the χ^2^ test with the appropriate number of degrees of freedom.

**Figure 2:**
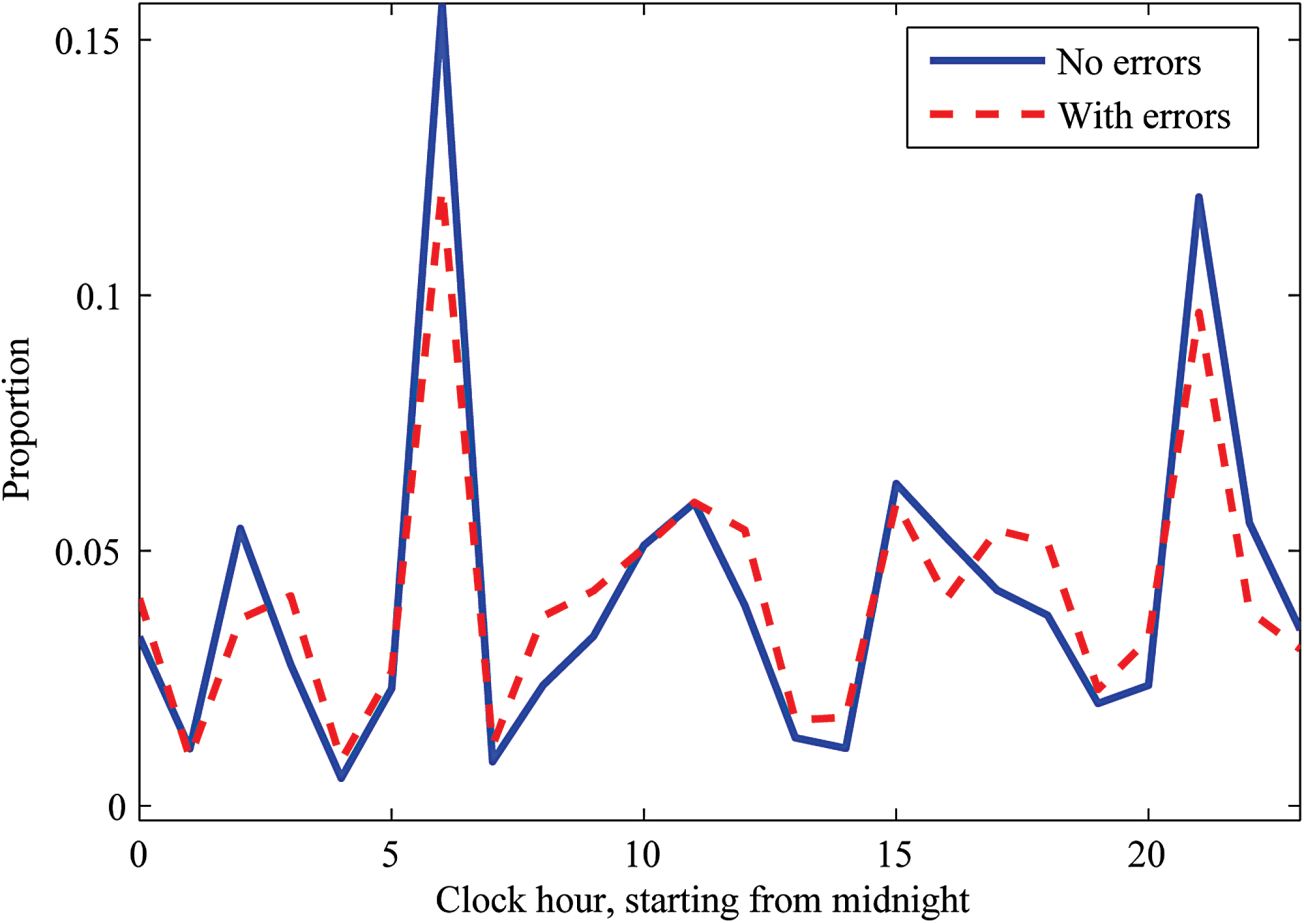
Distribution by hour of observation sets with (dashed line) and without (solid line) errors in assignment of scores to vital sign values, or errors in the aggregate score.

A complete observation set was defined as one which had (1) measurements for all five vital signs (figure 1), (2) weightings assigned to each vital sign or (3) an aggregate score. An observation set that should have generated an alert was defined as one for which (1) any of the vital signs which, when correctly weighted, would have resulted in a weight of 3, or (2) when the aggregate score, correctly calculated from the weights for individual recorded vital signs, would have equalled or exceeded a threshold of 4.

An observation set was defined as having an error in the assignment of weights if one or more of the weights assigned to the vital signs were incorrect. An observation set was defined as containing an error in the aggregate score if the aggregate score differed from the aggregate score that would have been obtained if (1) all weights assigned to the vital signs were correct, and (2) the summation of those vital sign weights was correct.

A sequence was defined as three or more consecutive complete observation sets with the same aggregate scores.

## Results

### Patients

200 upper-gastrointestinal surgical patients were included in the study, with a median age of 64 years (IQR 15 years), median length of stay of 10 days (IQR 7 days), and mortality of 3%. Full patient demographic data are provided in online supplementary appendix D.

### Completeness of observation sets and the effect on alerting rates

A total of 85.2% (14 313/16 795) of the observation sets contained measurements of all five vital signs. A total of 77.9% (13 079/16 795) of the observations sets contained measurements of all five vital signs and fully-recorded weightings. In total 65.5% (10 995/16 795) of all observation sets were complete and had a correctly calculated aggregate score. Temperature was the most commonly missing vital sign, being absent in 11.4% (1915/16 795) of observation sets. All others were recorded in more than 97% of observation sets.

Table 1 shows the breakdown of recorded scores based on whether they would lead to an alert or not, compared to the expected action (alert or no alert) for correctly calculated scores based on the same vital signs data. These are shown for 13 079 complete and 3716 incomplete observation sets as described above, and for the total of 16 795 sets.

**Table 1:** Observation sets according to alerting status

		Observation set					
Expected action by staff	Observed action by staff	Complete*		Incomplete		Total	
Correctly calculated score would lead to an alert	Staff record a score that would lead to an alert	696	(5.3%)	343	(9.2%)	1039	(6.2%)
Correctly calculated score would NOT lead to an alert	Staff record a score that would NOT lead to an alert	11 969	(91.5%)	3038	(81.8%)	15 007	(89.4%)
Correctly calculated score would NOT lead to an alert	Staff record a score that would lead to an alert	116	(0.9%)	118	(3.2%)	234	(1.4%)
Correctly calculated score would lead to an alert	Staff record a score that would NOT lead to an alert	298	(2.3%)	217	(5.8%)	515	(3.1%)
Total		13 079	(100%)	3716	(100%)	16 795	(100%)

*Complete for measurements of all five vital signs and fully recorded weightings.

EWS, early warning score.

A total of 33.1% (515/1554) of the observation sets that should have led to an alert did not have an alerting score recorded.

Only 18.4% (234/1273) of the observation sets recorded as causing an alert did not have physiological observations that should have led to an alert.

Incomplete data sets were more likely than complete data sets to contain observations that should have led to an alert (15.1% (560/3716) vs 7.6% (994/13 079), p<0.001), but less likely to have an appropriate alerting aggregate score recorded (61.3% (343/560) vs 70.0% (696/994), p<0.001).

Observation sets that should not have led to an alert but which had an alerting aggregate score recorded were more common in incomplete than complete observation sets (3.2% (118/3716) vs 0.9% (116/13 079), p<0.001).

### Time of day

The distribution of complete and incomplete observation sets by hour of day is shown (figure 2).

### Scoring patterns and incidence of error

Table 2 shows the 13 079 complete observation sets divided according to whether these sets: (1) formed part of a stable series; (2) occurred after the end of a stable series; or (3) were neither part of a stable series, nor occurred after a series (other). When leaving a series in which the aggregate scores were 0, observation sets were mis-scored more commonly than within the series, 55.3% (389/704) vs 3.0% (219/7332, p<0.001). For series in which the aggregate scores were >0, the subsequent observation set was mis-scored at a similar rate (27.9%, 83/298) to the error rate within the series (27.8%, 308/1108), p=0.988.

**Table 2:** Occurrence of observation sets with and without errors by observation sequence type

	Without errors n (%)		With errors n (%)	
Occurring as part of a stable series				
Within sequence where EWS values=0	7113	(97.0)	219	(3.0)
Within sequence where EWS values≥1	800	(72.2)	308	(27.8)
Occurring after the end of a stable series				
First observation after a sequence where EWS values=0	315	(44.7)	389	(55.3)
First observation after a sequence where EWS values≥1	215	(72.1)	83	(27.9)
Other	2424	(66.6)	1213	(33.4)
Total	10 867	(83.1)	2212	(16.9)

EWS, early warning score.

### Types of error in complete observation sets

Only 16.2% (2114/13 079) of the complete observation sets included one or more errors in the assignment of weights to vital sign measurements. 15.9% (2084/13 079) of the complete sets had an error in the aggregate score. Overall, 16.9% (2212/13 079) of the complete observations sets had errors in the assignment of weights, the aggregate score, or both.

Table 3 shows the complete observation sets by error type related to the correct alert status of the next observation set.

**Table 3:** Observation type by next observation set

Observation type	Next set n (%)					
	Alerting		Non-alerting		No next set	
Error with incorrect alert	17	(14.7)	99	(85.3)	0	(0)
Error-free no alert	431	(4.2)	9754	(94.4)	143	(1.4)
Error with incorrect no alert	78	(26.2)	218	(73.2)	2	(0.7)
Error-free alert	292	(54.2)	247	(45.8)	0	(0)
Error but correct alert	92	(58.6)	65	(41.4)	0	(0)
Error but correct no alert	118	(7.2)	1510	(92.0)	13	(0.8)
Total	1028	(7.9)	11 893	(90.9)	158	(1.2)

Only 14.7% (17/116) observation sets that contained an error which resulted in an ‘incorrect’ alert were followed by an observation set that correctly resulted in an alert. In comparison, 4.2% (431/10 328) of error-free, non-alerting observation sets were followed by an observation set that correctly resulted in an alert (p<0.001).

A total of 73.2% (218/298) of observation sets which contained an error that resulted in ‘incorrectly’ not alerting were followed by observations that correctly did not result in an alert. In comparison, 45.8% (247/539) of error-free alerting observation sets were followed by observations that correctly did not result in an alert (p<0.001).

## Discussion

Although EWS systems have become a standard of care,^6^ understanding of many aspects of their performance remains poor. As far as we can tell, our study (containing more than 16 000 observation sets) of a well-established system in stable clinical practice, is the largest assessment of errors within a paper-based track and trigger system undertaken to date. The scale of the study allowed us to investigate behaviour around ‘errors’ in novel ways.

In our study, 65.5% of all observation sets contained all five vital signs and had the correct aggregate EWS calculated, comparing favourably with a previous study where only 54.4% of all observation sets met these criteria (and only four vital signs were required).^1^ However, our results are far from reassuring. Almost a third of observation sets where an alerting aggregate score should have been generated did not have a score recorded. One in six observations sets in which an alerting aggregate score was recorded did not contain vital signs that should have generated an alert. In both cases, errors were much more likely when the observation set was incomplete. As incomplete observation sets were also particularly likely to contain vital signs that should have led to an alerting aggregate score, errors in incomplete observation sets are particularly likely to affect patient care. Decreasing the number of incomplete observation sets would seem a relatively easy target for improvement.

Interestingly, though staffing levels were somewhat lower at night, error rates were not increased, in contrast to previous findings.^7^ This may be because the ward studied is staffed to reflect a relatively high nursing workload. The usual practice of staff undertaking two ‘observations rounds’ per day is shown by the two peaks in figure 2, similar to those previously reported.^8^

Errors of weighting and errors of summation featured equally, with the large majority of observation sets with errors showing both weight assignment and calculation mistakes. At around 17% of all observation sets, the combined error rate is lower than reported by Edwards *et al*,^2^ where assignment errors predominated and the overall error rate was 36%.

Errors were particularly likely when a patient was first starting to become unstable (leaving a sequence of zero scores), where more than 50% of the first >0 aggregate scores were mis-scored. An opportunity to recognise early deterioration is being missed. The fact is that a preceding stable aggregate score sequence affects the ability to identify the first signs of instability and this could be handled electronically by only allowing the clinician to see the previous observation sets once all the vital signs had been entered. However, this approach may not be necessary if assignment and summation of weights were automated.

Perhaps the most important discovery in this paper is that the ‘errors’ are not all that they first appear to be. For both observation sets that incorrectly resulted in an alert and observation sets that incorrectly did not result in an alert, the subsequent observation set was disproportionately more likely to have the same alert status as the preceding ‘incorrect’ set than if the ‘error’ were not present. It appears that clinicians either use additional information with that available from the vital signs of the patients^9^ or sense information within the measured vital signs that the EWS system does not encapsulate in their overall assessment of a patient's risk status. It is clear that they detected both deterioration and improvement in advance of the EWS system. If these ‘correctly predictive’ aggregate scores are taken as the optimum assessment of patient status, the effects on ‘error’ rates are dramatic, reducing the proportion of complete observation sets that should have had an alerting aggregate score but did not from 30.0% (298/994, table 1) to 8.0% (78/994, table 3). Why is this information so critical? In our efforts to exclude all ‘error’, as defined by the mathematically correct aggregate score for a particular observation set, we must not risk losing the information that allows clinicians to outperform the EWS system. There is a danger that the ‘power of the score’ will subjugate both clinicians’ concern and the willingness of the response team to respond to a patient who is physiologically ‘normal’ as defined by the EWS criteria. Recording a mathematically incorrect aggregate score is clearly not the optimal approach. Although a ‘clinician concern’ option was present in our EWS system, this concern was not assigned a weight and hence, did not contribute to the aggregate score. Assigning ‘clinician concern’ a weight to form part of the overall aggregate score is an approach that may have merit.

Our work has limitations. First, it was restricted to a single surgical ward, with EWS training specific to the particular institution. Different error behaviours may occur in different environments, with different training systems. Staff were aware that they were taking part in a study, which may have affected overall error rates. As recent work suggests that errors in chart analysis may depend on chart design,^10^ some of our findings may be restricted to the particular chart used during the study. It is possible that our researchers’ interpretation of the vital sign values recorded graphically on the chart could lead to incorrect error rates, a risk inherent in all such analyses of paper charts. However, the rules we adopted, combined with blind double data entry and third researcher resolution should have minimised this risk. We believe that this is an appropriate approach when undertaking research regarding error rates with paper-based EWS systems. Importantly, our major findings, relating to patterns surrounding ‘errors’ are unlikely to be affected by misinterpretation of individual original recordings.

What can be learnt from our study about the assessment of local EWS performance? A large majority of observation sets score zero (reducing the opportunity for error), and so clinically important error rates are obscured by reporting overall values. Error rates concerning vital signs that would lead to a change in patient care are much greater than overall error rates. Assessments of EWS performance should, therefore, report the proportions of alerts missed and erroneously generated, along with a measure of the error rate in recognising when a patient first becomes unstable. These are the errors most likely to affect patient outcomes, but are not captured in current national audit recommendations.^11^

What can be done beyond local performance review to improve the recognition of deteriorating patients? Some information is already available. Simpler scores result in lower error rates.^5^ However, simpler scores would need to have equivalent capacity to detect deterioration and avoid unnecessary alerting. Designing a chart by taking human factors into account can reduce interpretation errors,^10^ ^12^ such as those demonstrated by our transcribers. It may be that this approach can also improve error rates in weight assignment and aggregation. To these, our study findings add that incomplete observation sets should be avoided, as these are consistently associated with missing important changes in a patient's condition.

Most importantly, observation sets that ‘incorrectly’ alert or ‘incorrectly’ do not alert are predictive of the next observation set. By detecting information not currently used within early warning systems in their overall assignment of a patient’s risk status, clinicians detect both deterioration and improvement in advance of the early warning system. Work is urgently needed to understand if and how this information can be captured, especially as there is a trend towards automated measurement of vital signs, electronic assignment of a weight to vital sign values, and automated aggregation of weightings to generate an overall score.

## Conclusion

Errors are much more common in observation sets which should lead to a change in patient care than in those that should not. Therefore, assessments of EWS performance should report the proportions of alerts missed and erroneously generated. Errors that lead to a failure to recognise when a patient first becomes unstable should also be reported.

Incomplete observation sets should be avoided, as these are more likely to contain vital signs that should lead to an alert and more likely to be associated with errors that cause an alert not to be generated.

Clinicians are able to outperform the EWS. Understanding how they do this and how to incorporate this ability into future EWSs are important areas for future research.
